# Implementation of an automated early warning scoring system in a surgical ward: Practical use and effects on patient outcomes

**DOI:** 10.1371/journal.pone.0213402

**Published:** 2019-05-08

**Authors:** Eveline Mestrom, Ashley De Bie, Melissa van de Steeg, Merel Driessen, Louis Atallah, Rick Bezemer, R. Arthur Bouwman, Erik Korsten

**Affiliations:** 1 Department of Anaesthesiology, Catharina Hospital, Eindhoven, The Netherlands; 2 Patient Care & Measurements, Philips Research, Eindhoven, The Netherlands; 3 Department of Electrical Engineering, Eindhoven University of Technology, Eindhoven, The Netherlands; University Magna Graecia of Catanzaro, ITALY

## Abstract

**Introduction:**

Early warning scores (EWS) are being increasingly embedded in hospitals over the world due to their promise to reduce adverse events and improve the outcomes of clinical patients.

The aim of this study was to evaluate the clinical use of an automated modified EWS (MEWS) for patients after surgery.

**Methods:**

This study conducted retrospective before-and-after comparative analysis of non-automated and automated MEWS for patients admitted to the surgical high-dependency unit in a tertiary hospital. Operational outcomes included number of recorded assessments of the individual MEWS elements, number of complete MEWS assessments, as well as adherence rate to related protocols. Clinical outcomes included hospital length of stay, in-hospital and 28-day mortality, and ICU readmission rate.

**Results:**

Recordings in the electronic medical record from the control period contained 7929 assessments of MEWS elements and were performed in 320 patients. Recordings from the intervention period contained 8781 assessments of MEWS elements in 273 patients, of which 3418 were performed with the automated EWS system. During the control period, 199 (2.5%) complete MEWS were recorded versus 3991 (45.5%) during intervention period. With the automated MEWS systems, the percentage of missing assessments and the time until the next assessment for patients with a MEWS of ≥2 decreased significantly. The protocol adherence improved from 1.1% during the control period to 25.4% when the automated MEWS system was involved. There were no significant differences in clinical outcomes.

**Conclusion:**

Implementation of an automated EWS system on a surgical high dependency unit improves the number of complete MEWS assessments, registered vital signs, and adherence to the EWS hospital protocol. However, this positive effect did not translate into a significant decrease in mortality, hospital length of stay, or ICU readmissions. Future research and development on automated EWS systems should focus on data management and technology interoperability.

## Introduction

Automated early warning score (EWS) systems are increasingly embedded in clinical practice to improve registration and awareness of vital signs and enhance rapid response teams (RRT) notifications[1]. The impact on clinical outcomes of these systems remains uncertain in various populations, for example in the high-risk surgical population, which is the focus of this study.

Complications that are frequently encountered on the general ward can lead to major adverse events such as unplanned intensive care unit (ICU) admissions, cardiorespiratory arrest, and mortality[2, 3]. The EWS has been developed as an objective bedside tool to help clinicians identify patients at risk of adverse events[4, 5]. Since its introduction in the late nineties, studies have shown varied results on the predictive value of EWS as well as its implications on clinical outcomes[4, 6–9]. Consequently, newer versions of EWS have been developed to improve clinical relevance, such as the modified early warning score (MEWS)[10]. Digital automated systems have been introduced to assist clinicians in completing the EWS assessment. Several studies emphasized that these systems provide a faster completion of EWS with increased accuracy. This is important since complete and accurate registration on a regular base is essential for the effectiveness of EWS, especially when upcoming assisting medical technologies, such as clinical decision support tools, rely on these data[1, 11–16].

In addition, many studies have shown that automated systems reduce mortality and length of hospital stay. These studies also showed an improvement in the survival of patients treated by RRTs after EWS-triggered notification[1, 11, 17, 18]. On the other hand, the study of Dawes et al. showed no significant improvement in mortality among 3184 patients admitted to an acute medical unit after an electronic alerting physiological scoring system was introduced[19].

The automation of MEWS systems may be more effective for specific subgroups of patients such as high-risk surgical patients on the ward since postoperative complications develop more often and documentation on vital signs is known to be lacking there[20, 21].

The aim of this retrospective before-and-after cohort study is to investigate whether an automated MEWS system on a surgical high dependency unit (HDU) had a positive effect on clinical practice, in terms of improved documentation of vital signs and complete EWS assessments, and EWS protocol adherence. Secondary aim of the study was to evaluate the impact on clinical outcomes, such as mortality, length of stay, and ICU readmissions.

## Methods

### Study design

This is a retrospective before-and-after comparative analysis of clinical practice and outcomes for patients admitted to the eight-bed surgical HDU in the Catharina Hospital, a tertiary teaching hospital in Eindhoven, the Netherlands. This unit functions as a step-down unit between the ICU and the regular surgical ward in the postoperative phase after major elective and acute surgeries. These surgeries include major gastro-intestinal, oncologic, and vascular surgeries, such as pylorus preserving pancreaticoduodenectomy, open vascular aortic surgery and hyperthermic intraperitoneal chemotherapy.

Inclusion criteria were patients admitted for step-down care after surgery, who required an ICU admission for postoperative hemodynamic surveillance. Exclusion criteria were patients under 18 years old, ICU admission for other reasons than hemodynamic surveillance, such as electrolyte surveillance after thyroidectomy, and patients who had a second admission on the HDU after a hospital discharge during the study period or who were admitted for other reasons than step-down care.

The study period consisted of two 15 month phases: the control phase (January 2012 until March 2013) and the intervention phase (June 2013 until August 2015). Data from a three-month period between the phases was omitted to account for any influences of the training period directly after implementation of the automated MEWS system, the Philips IntelliVue Guardian Solution (Guardian). The study was approved by the Medical research Ethics Committees United (MEC-U; study ID:non-WMO 2015–87). The study was classified as non-WMO by the MEC-U based on the retrospective design. Therefore obtaining an informed consent was not deemed necessary as it conforms to the Dutch Agreement on Medical Treatment Act. All data were analyzed anonymously.

#### Control period

In correspondence with common daily practice, the MEWS was used during the control period. This MEWS was introduced in 2011 in the hospital during a national study by Ludikhuize et al (Table 1) [17]. MEWS parameters are heart rate, oxygen saturation (SpO_2_), respiratory rate, non-invasive blood pressure, temperature, AVPU scale, and 24-hour urine production. According to the MEWS hospital protocol, these parameters were assessed at bedside within certain timeframes based on the previous MEWS (Table 1). All measured parameters were manually recorded in the electronic medical record (EMR). Nurses could manually calculate the MEWS using cards containing the MEWS algorithm. They were instructed to alert the physician on call in case of a MEWS ≥3 for assessment and potential treatment (Table 2). Nurses could also escalate to call the RRT of the ICU directly if the physician on call were not available or if initiated treatments after a certain MEWS did not lead to any improvement.

**Table 1:** Modified early warning score system.

Score	3	2	1	0	1	2	3
**Heart rate (beats/min)**		<40	40–50	51–100	101–110	111–130	>130
**Systolic blood pressure (mm Hg)**	<70	70–80	81–100	101–200		>200	
**Respiratory rate (breaths/min)**		<9		9–14	15–20	21–30	>30
**Temperature (°C)**		<35.1	35.1–36.5	36.6–37.5	>37.5		
**Level of consciousness**				A (Alert)	V (Voice responsive)	P (Pain responsive)	U (Unconscious)
**Worried** about patient’s condition: 1 point							
**Urine production** below 75ml during previous 4h : 1 point							
**Oxygen saturation** below 90% despite adequate oxygen therapy: 3 points							

**Table 2:** Response system for modified early warning score.

MEWS	Time till next MEWS assessment
**0**	Next shift (within 24 hours)
**1**	Within 8 hours
**2**	Within 4 hours
**3**	Within 1 hour; Consult responsible physician
**≥ 4**	Within 1 hour; Consult responsible physician and consider to consult RRT

#### Intervention period

The electronic EWS system Philips IntelliVue Guardian Solution (Guardian) was implemented on the HDU over a three-month period between the control and intervention phase. This system facilitated the acquisition of vital signs and the completion of MEWS to provide automated clinical decision support and awareness to the nursing staff. The device consisted of two spot-check monitors which were taken to the bedside of the patients to measure respiratory rate, non-invasive blood pressure, heart rate and SpO_2_. Urine output, level of consciousness (AVPU scale), temperature, and the nurse’s level of concern were manually entered in the Guardian software. The device calculated the MEWS values and showed them on the screen of the device as well as on a monitor at the central nurse station. In addition to the MEWS value, a short advice was displayed on the screen for further monitoring, such as the recommended time until the next assessment, or recommended actions, such as alerting a physician or the RRT. In addition, the monitor at the central nurse station also displayed these features. Every spot-check observation was stored in a database, which was not connected to the electronic medical record (CS-EZIS test, Chipsoft BV, Amsterdam, The Netherlands). Nurses were required to copy the measured vital parameters and MEWS into the EMR. All nurses received appropriate training on data collection before implementation of the Guardian system. All parameters needed to be recorded within a 15-minute timeframe to be considered in a single MEWS measurement. During the intervention period, the conventional methods used during the control period were still available to collect MEWS parameters and record them in the EMR, and although nurses were trained to use the automated system, they were free to work according to their preference.

### Data collection and outcomes

The following data were retrieved from the EMR or the Guardian database during the periods: patient characteristics, vital signs and all other elements from the MEWS, and outcomes such as ICU re-admission, mortality, and length of stay. APACHE II, APACHE IV and SAPS II scores were collected during the first postoperative admission at the ICU or in the event of an ICU readmission. The results of this study were divided into two categories: operational outcomes (primary outcomes) and clinical outcomes (secondary outcomes).

#### Primary outcomes

Operational outcomes under study include the practical clinical use of the automated EWS system, such as the number of documented MEWS elements and calculated MEWS values recorded in the EMR. In addition, the percentage of complete MEWS, daily patterns of the assessments, and the time interval between assessments were calculated. Time interval between assessments was also used to see if the next assessment was performed conform MEWS hospital protocol (Table 1). Measurements with a time interval of less than 15 minutes were considered a single assessment.

#### Secondary outcomes

The clinical outcomes analyzed include the impact of the use of the automated MEWS system on the length of stay in the hospital, in-hospital and 28-day mortality, and ICU readmission rate. An ICU readmission was defined as a transfer to a higher level of care, such as ICU, medium care, or cardiac care unit.

Sub-analysis was performed for readmitted patients at the ICU; APACHE II, APACHE IV, and SAPS II scores on admission to the ICU were compared between the control and intervention group. Other outcomes for this subgroup analysis were the length of stay at the ICU and ICU mortality. In addition, a similar subgroup-analysis was performed for different age groups (≤49 years, 50–69 years and ≥70 years) or if certain patient characteristics differed significantly between the control and intervention period.

### Statistical analysis

All statistical analyses were performed using IBM SPSS Statistics version 23 (IBM Corp, Armonk, New York, released 2015) [22]. Normality of the data sets was tested using a Kolmogorov-Smirnov test. Continuous data are presented as means and standard deviations or medians and interquartile data based on the distribution of the data. Categorical data are presented as proportions or percentages. The Mann-Whitney test was used to test for differences in continuous variables with non-normal distributions and the chi-square test Fisher’s exact test was used to test for differences in categorical groups. A value of p<0.05 was considered statistically significant.

## Results

A total of 594 patients were included for analysis, 320 patients for the control group and 274 patients for the intervention group. Both groups were comparable in terms of age, gender, and APACHE II and -IV scores at the time of first ICU admittance postoperatively (Table 3). The control group consisted of significantly less patients undergoing oncologic abdominal surgery (69.7 versus 80.3%; p = 0.01) and more patients undergoing aortic surgery (20.0 versus 12.4%; p 0.01).

**Table 3:** Baseline characteristics of all included patients (A) and patients readmitted to ICU (B).

**A. Patients overall**	**Control group**	**Intervention group**	**p-value**
Number of patients	320	274	
Age in years, median (IQR)	67 (15)	67 (16)	0.29
Male gender, number (%)	204 (63.8)	173 (63.1)	0.88
Unplanned (acute) surgery (%)	57 (18.1)	37 (13.5)	0.13
Type of surgery			0.01
- Abdominal, oncology (%)	223 (69.7)	220 (80.3)	
- Abdominal, benign (%)	33 (10.3)	20 (7.3)	
- Vascular, aortic (%)	64 (20.0)	34 (12.4)	
APACHE II first ICU admission, median (IQR)	14 (6)	14 (5)	0.35
APACHE IV first ICU admission,median (IQR)	37 (15)	38 (17)	0.39
SAPS II first ICU admission, median (IQR)	31 (15)	31 (17)	0.44
**B. Patients readmitted ICU**	**Control group**	**Intervention group**	**p-value**
Number of patients	43	29	
Age in years, median (IQR)	65 (19)	67 (10)	0.30
Male gender, number (%)	33 (76.7)	23 (79.3)	0.80
Unplanned (acute) surgery (%)	8 (18.6)	4 (13.8)	0.83
Type of surgery			0.17
- Abdominal, oncology (%)	29 (64.4)	25 (86.2)	
- Abdominal, benign (%)	8 ((18.6)	2 (6.9)	
- Vascular, aortic (%)	6 (14.0)	2 (6.9)	
APACHE II, median (IQR)			
- first ICU admission	14 (4)	16 (6)	0.02
- ICU readmission	20 (11)	19 (8)	0.35
APACHE IV, median (IQR)			
- first ICU admission	37 (13)	43 (30)	0.08
- ICU readmission	52 (50)	52 (20)	0.55
SAPS II, median (IQR)			
- first ICU admission	29 (9)	33 (23)	0.18
- ICU readmission	40 (24)	38 (21)	0.46
ICU interventions, number (%)			
- Arterial line	32 (74.4)	21 (72.4)	0.85
- Vasopressor use	16 (37.2)	11 (37.9)	0.95
- Mechanical ventilation	29 (67.4)	14 (48.3)	0.10

### Operational outcomes

**Fig 1:**
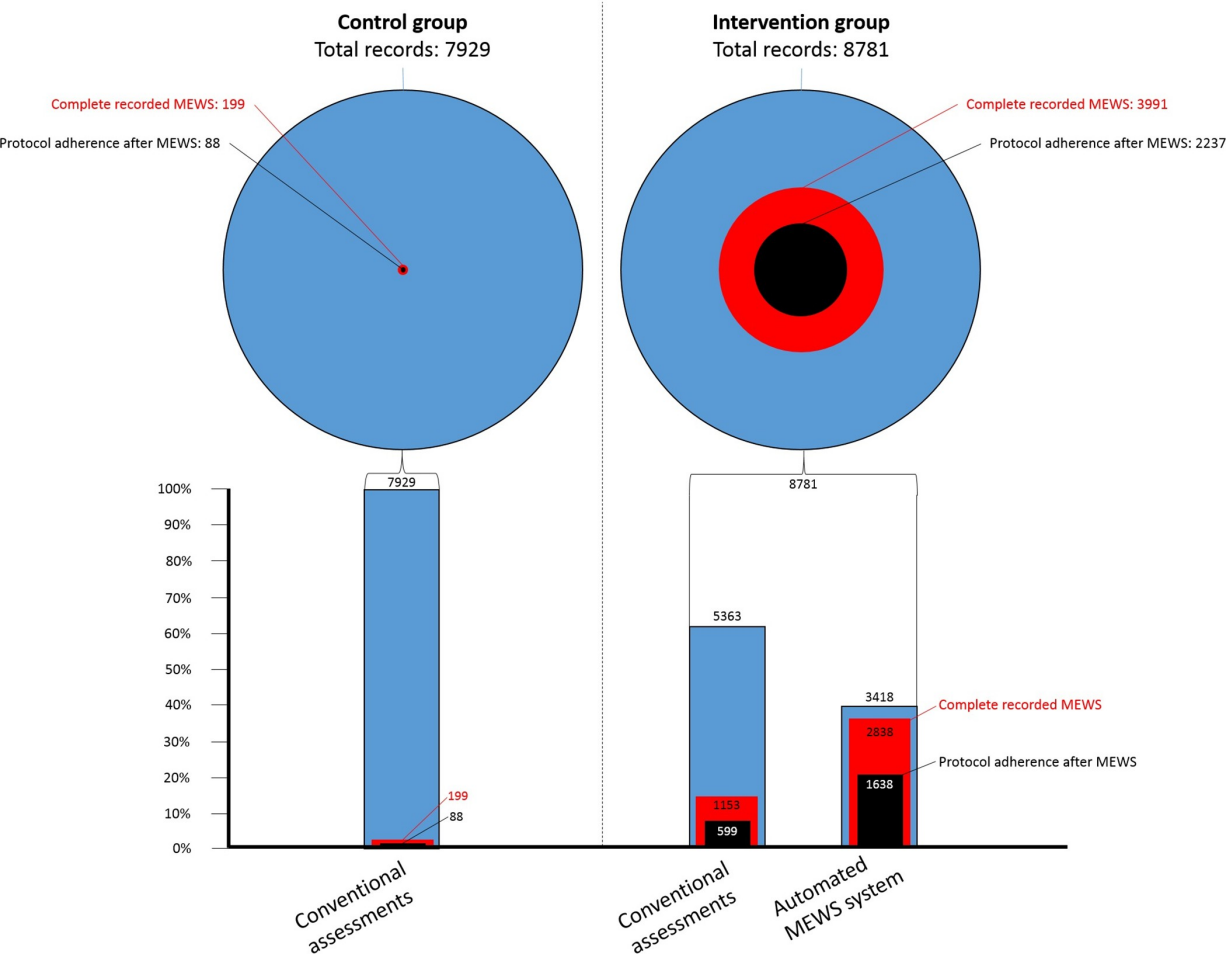
MEWS protocol adherence. The number of complete recorded MEWS and protocol adherence according to these MEWS for the control period and the intervention period, subdivided in conventional and automated MEWS assessments during the intervention period.

During the control period, a total of 7929 records of one or more elements of the MEWS were retrieved from the EMR. During the intervention period the total number of recorded assessments was 8781. Of these 8781 assessments, 3418 (39%) were recorded with the automated MEWS system. The other 5363 (61.1%) assessments were recorded with conventional monitoring systems. Results are shown in Fig 1.

The adherence to MEWS hospital protocol improved when the automated MEWS system was involved, from 1.1% (88 of 7929 assessments) in the control group to 25.4% (2237 of 8781 assessments) in the intervention group. Within the intervention group, the adherence to MEWS hospital protocol improved from 10.8% (599 of 5363 assessments) when a conventional method was used to 47.9% (1638 of 3418 assessments) when the automated MEWS system was used (Fig 1).

**Fig 2:**
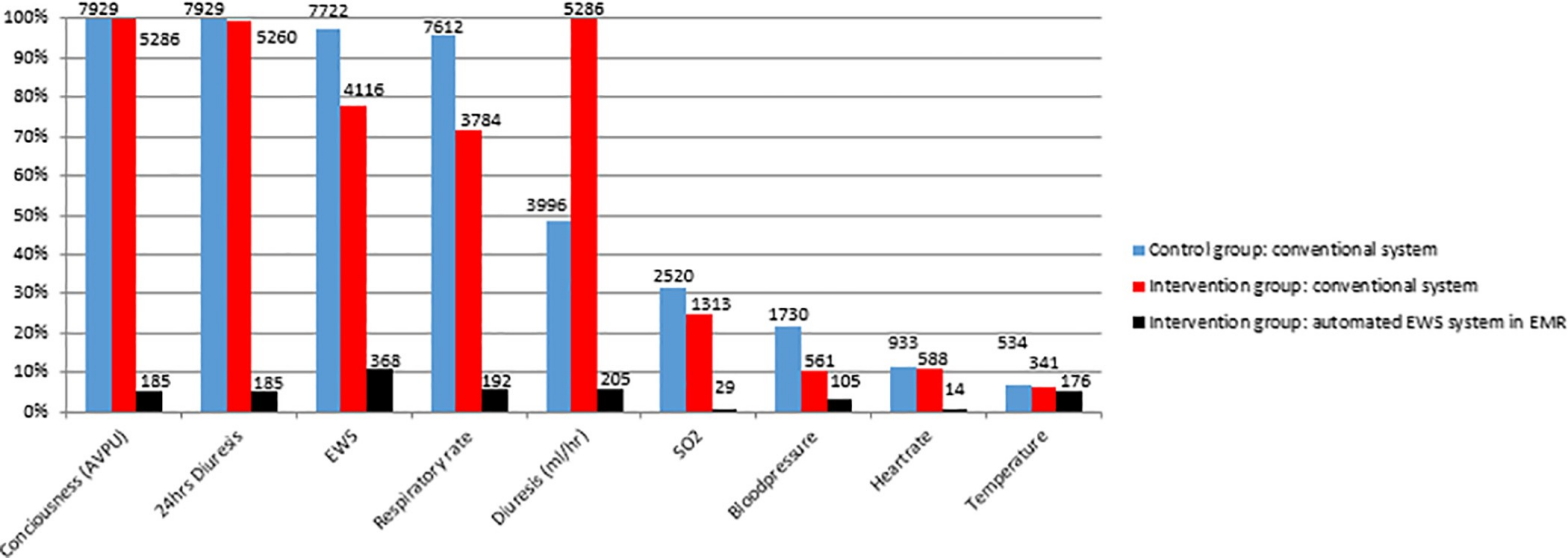
Percentages and absolute numbers of missing MEWS elements assessments.

**Fig 3:**
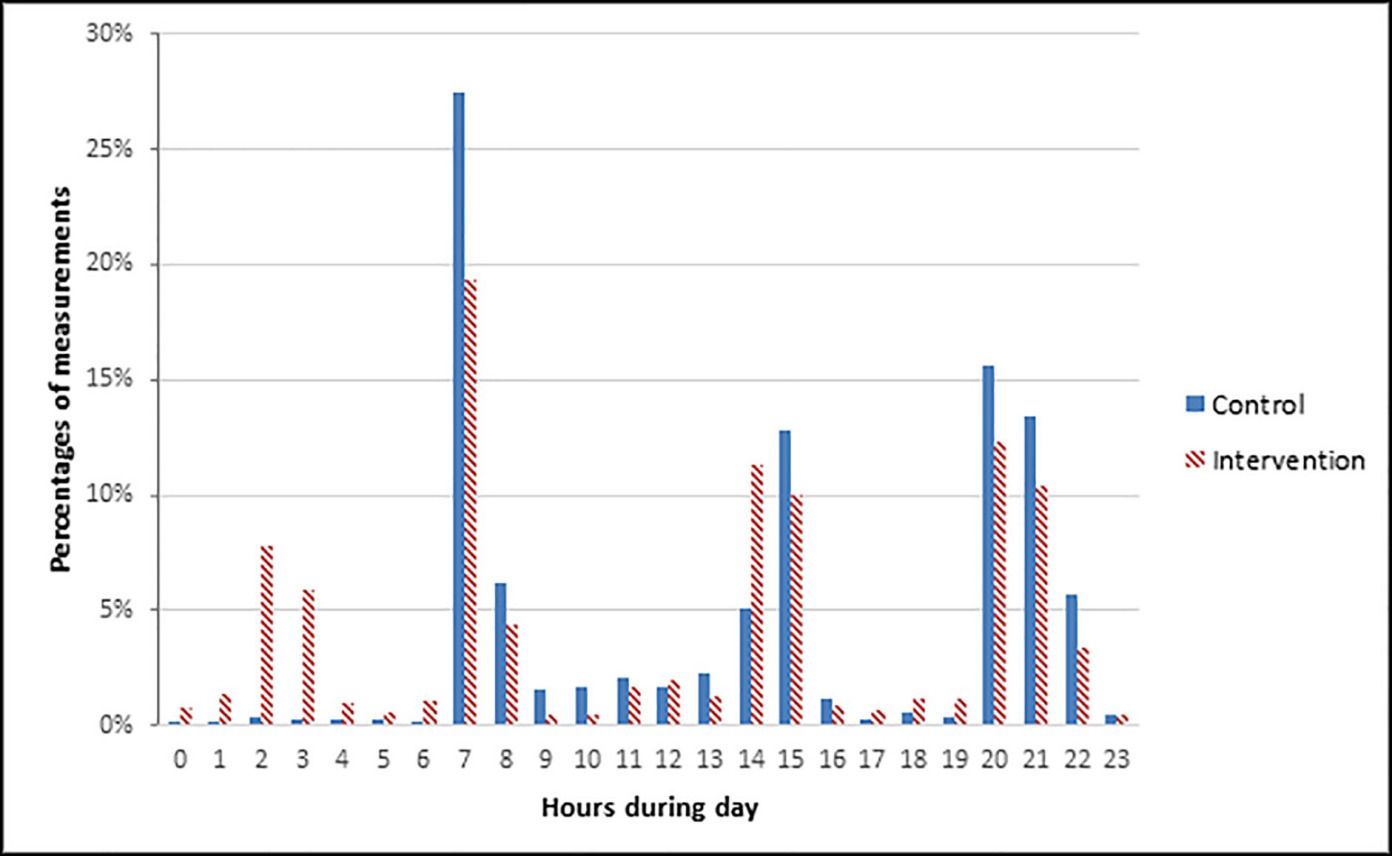
The 24-hour pattern of MEWS assessments. Pattern for control and intervention group.

The implementation of the automated MEWS system resulted in significantly more completed MEWS containing all the MEWS elements from 199 (2.5%) complete assessments in the control group to 3991 (45.5%) complete assessments in the intervention group (p<0.001). The number and percentage of missing elements in the MEWS for each documented record is shown in Fig 2. The most pronounced difference was observed for respiratory rate (96% versus 3%) and level of consciousness (100% versus 3%). The daily pattern is shown in Fig 3 and was comparable in both groups with three peaks during 24 hours corresponding to the daily nursing rounds. The median time until the next assessment was significantly shorter when a MEWS of 2 and higher was measured with the automated MEWS system compared to the conventional method between both groups and within the intervention group (Table 4.)

**Table 4:** Median time in hours until next assessment.

**MEWS**	**Control period**		**Intervention period**		**p value**	**Z-score**
	Conventional system		Conventional and automated EWS system			
	N	Median hours (IQR)	N	Median hours (IQR)		
**0**	33	7.40 (4.1)	668	6.06 (3.49)	0.011	-2.539
**1**	59	6.02 (5.46)	1370	5.86 (3.22)	0.35	-0.931
**2**	42	6.66 (4.32)	1144	5.68 (3.42)	0.011	-2.539
**3**	65	5.10 (6.79)	809	4.62 (3.21)	0.019	-2.338
**MEWS**	**Intervention period**		**Intervention period**		**p value**	**Z-score**
	Conventional system		Automated EWS system			
	N	Median hours (IQR)	N	Median hours (IQR)		
**0**	186	6.01 (4.72)	482	6.09 (3.09)	0.36	-0.922
**1**	388	5.78 (4.00)	982	5.95 (3.01)	0.36	-0.914
**2**	328	6.08 (3.72)	816	5.55 (3.44)	0.002	-3.037
**3**	251	5.06 (4.60)	558	4.29 (5.30)	<0.001	-3.997
**MEWS**	**Control period**		**Intervention period**		**p value**	**Z-score**
	Conventional system		Automated EWS system			
	N	Median hours (IQR)	N	Median hours (IQR)		
**0**	33	7.40 (4.11)	482	6.09 (3.09)	0.014	-2.467
**1**	59	6.02 (5.46)	982	5.95 (3.01)	0.4	-0.838
**2**	42	6.66 (4.32)	816	5.55 (3.44)	0.004	-2.879
**3**	65	5.10 (6.79)	558	4.29 (5.30)	0.003	-2.992

### Clinical outcomes

There were no significant differences in outcomes on mortality or length of stay (Table 5). In addition to that, the number of readmitted patients at the ICU and their severity of illness at readmission based on the SAPS II, and APACHE II and–IV did not significantly differ between the control and the intervention group.

**Table 5:** Clinical outcomes of all included patients (A) and patients readmitted to ICU (B).

**A. Patients overall**	**Control group**	**Intervention group**	**p-value**
Unplanned (acute) surgery (%)	57 (18.1)	37 (13.6)	0.16
Length of stay days, median (IQR)			
- Hospital	12 (10)	11 (8)	0.39
- ICU previous to HDU	1.1 (1.0)	1.1 (1.0)	0.07
- HDU	7.5 (7.0)	7.1 (6.0)	0.59
Mortality, number (%)			
- In-hospital	5 (1.6)	3 (1.1)	0.9
- 28-day	7 (2.2)	2 (0.7)	0.27
Readmission ICU, number (%)	43 (13.4)	29 (10.6)	0.36
**B. Readmitted patients**	**Control group**	**Intervention group**	**p-value**
Length of stay days, median (IQR)			
- Hospital	25 (43)	28 (26)	0.55
- ICU previous to HDU	1 (1.1)	1.1 (1.0)	0.14
- HDU	3.3 (4.2)	2.4 (4.8)	0.32
- ICU readmission	3.44 (9.3)	3.7 (3.6)	0.57
Mortality, number (%)			
- In-hospital	4 (9.3)	2 (6.9)	1.0
- 28-day	5(11.6)	0 (0)	0.15

Subgroup-analyses of three age groups (≤49 years, 50–69 years and ≥70 years) or per type of surgery did not result in significant differences between groups.

## Discussion

The results of this retrospective study in a surgical high-dependency unit show that the use of an automated MEWS system improves the recording separate MEWS elements and complete MEWS assessments, as well as the resulting adherence to the MEWS hospital protocol. The use of this system improved the MEWS hospital protocol adherence for MEWS assessments using conventional methods during the intervention period. After implementing the automated MEWS system, 39% of the assessments were performed during the intervention period. Although there was a trend towards improved clinical outcomes in this period, this study did not show significant differences in mortality, length of stay, ICU readmission rate, or severity of illness at ICU readmission.

Similar to other studies, our study demonstrated improved accuracy and completeness in the recording of vital signs and complete MEWS after implementation of an automated MEWS system[14, 23]. These positive results on registration outcomes are important since valuable, reliable data become available for research, as quality of care indicators, and as input for clinical decision support system to improve clinical attendance and patient survival [1, 11, 13–16, 23, 24].

In contrast to similar studies, however, this study did not find a significant improvement of clinical outcomes[1, 11, 13, 14]. The most important difference between this work and others who have found such improvement was the practical and logistical use of the automated MEWS system. The implementation and data management of the automated MEWS in this study was different from previous studies[1, 13–16, 24]. For example, in this study, the automated MEWS system functioned as a CDSS (clinical decision support system) on the bedside and results needed to be copied manually into the hospital EMR system. The lack of direct integration with the EMR and also use of the conventional method during the intervention period were in contrast to previous studies where data collection changed from paper records during the control period to digital recording with automated EWS for the intervention period. In addition, some previous studies applied automated EWS assessments to provide clear overviews with trends on big screens or connections to beepers of ward physicians and RRT [1, 13–16, 24]. These differences in integration of automated EWS systems in daily clinical practice might explain the differences in clinical outcomes.

Second, several previous studies vary in methods used to assess the EWS, like single parameter scoring, EWS, and MEWS[1, 11, 13, 14]. Consequently, this heterogeneity prevents adequate comparison of clinical outcomes.

Third, the present study focused on non-elderly, high-risk surgical patients, while previously studied populations were mainly performed on general surgical and medical wards, and found positive effects in the elderly[1, 11, 13, 14].

Fourth, the difference in outcomes might be due to the variation in study design. Observation periods between studies differed and could have led to educational or Hawthorne effects in studies with relatively short intervention periods [11, 14]. Additionally, prospective research may be more prone for higher acuity or over-triage of less severe ill patients for ICU admission, possibly leading to diversion of real practice during the intervention periods. For example, the largest prospective multi-center study reporting positive clinical outcomes of Bellomo et al. readmitted more patients to the ICU while the need for ICU interventions like vasopressors, arterial lines and mechanical ventilation in their intervention period was significantly lower compared to the control period[11]. Even though this is explained as a result of earlier recognition of the deteriorating patient, one should be aware that this might also be explained by over-triage. This retrospective study was not able to find significant differences in severity of illness, ICU interventions or clinical outcomes in the overall study population and ICU readmissions. Therefore, retrospective long term designed studies may provide important insights on the effect of automated EWS systems while preventing diversion of real practice [25].

The retrospective nature of this study in inherent for the limitations of having missing data. Assessments might have been performed without being recorded in the EMR or automated MEWS system. Another limitation is in the size of the groups analyzed. If the study were to be powered based on mortality alone, there would have been a need for increasing patient cohort size from approximately 500 to more than 10,000 patients. In addition to that, the administrative burden for the nursing staff can result in missing data due to the automated MEWS system lacking interoperability with the EMR and hospital’s computer server. This is likely to be an important reason why only a trends towards improved clinical outcomes was found in this retrospective real-life cohort study.

Automated EWS systems that provide more complete and accurate recording of data have a great potential for future clinical decision support systems and early deterioration detection. Especially if additional data is included such as laboratory, pharmaceutical, and historical data, in combination with the upcoming use of machine learning and artificial intelligence for department or patient-specific MEWS algorithms[26–30]. Therefore, interoperability between automated MEWS systems and the EMR or other medical devices seems essential to prevent solely data input without data management and valuable output to save time for medical staff while achieving more consistent improved clinical outcomes.

## Conclusion

Implementation of an automated MEWS on a surgical high dependency unit improves the number of complete MEWS, registered vital signs, and adherence to the local MEWS hospital protocol. However, this positive effect did not translate into a significant decrease in mortality, hospital length of stay, or ICU readmissions. Future research and development on automated EWS systems should focus on data management and technology interoperability to provide actionable insights to the right person at the right time.
